# Physical Activity during Winter in Old-Old Women Associated with Physical Performance after One Year: A Prospective Study

**DOI:** 10.1155/2015/253451

**Published:** 2015-06-22

**Authors:** Atsushi Mizumoto, Hikaru Ihira, Keitaro Makino, Shigeyuki Saitoh, Hirofumi Ohnishi, Taketo Furuna

**Affiliations:** ^1^Department of Physical Therapy, School of Health Sciences, Sapporo Medical University, South 1, West 17, Chuo-ku, Sapporo, Hokkaido 060-8556, Japan; ^2^Department of Nursing, School of Health Sciences, Sapporo Medical University, South 1, West 17, Chuo-ku, Sapporo, Hokkaido 060-8556, Japan; ^3^Department of Cardiovascular, Renal and Metabolic Medicine, Sapporo Medical University, South 1, West 17, Chuo-ku, Sapporo, Hokkaido 060-8556, Japan; ^4^Department of Public Health, Sapporo Medical University, South 1, West 17, Chuo-ku, Sapporo, Hokkaido 060-8556, Japan

## Abstract

*Background*. The aim of this study was to evaluate whether the decline of physical activity during winter influences physical performances (after 1 year) in old-old women. *Methods*. Fifty-three Japanese women (mean age: 78.4 ± 3.2 years) participated in this study. Data of physical activity was collected by using an accelerometer at baseline and 3-month follow-up, and participants who decreased step counts in this period were defined as declining groups. We measured grip strength, knee extensor strength, total length of the center of gravity, hip walking distance, and maximum walking speed to evaluate physical performances at baseline and 1-year follow-up. Repeated-measures analysis of variance determined the difference in physical performance between declining groups and maintenance group with maintained or improved step counts. *Results*. Daily step counts for 22 older women (41.5%) decreased during winter. A statistically significant interaction effect between group and time was found for maximum walking speed (*F*(1,50) = 5.23, *p* = 0.03). *Post hoc* comparisons revealed that walking speed in the maintenance group significantly increased compared with baseline (*p* = 0.01); the declining group showed no significant change (*p* = 0.44). *Conclusion*. Change of physical activity during winter influences the physical performance level after 1 year in community-dwelling old-old women, particularly its effect on maximum walking speed.

## 1. Introduction

Physical activity promotes physical and mental health, improves social bonds and quality of life, and helps prevent disease. In addition, it also provides economic benefits and contributes to environmental sustainability [1]. Globally, noncommunicable diseases account for approximately 60% of all deaths. Physical inactivity is a major risk factor associated with contracting noncommunicable diseases and is attributed to causing an estimated 3.2 million deaths per year [2]. Hence, increased physical activity is universally recommended for all age groups.

Key seasonal and climactic environmental factors such as temperature, precipitation, and snow affect physical activity levels. Several studies have reported that the range of activity among adults decreases during winter compared to that in summer [3–5]. Chan and Ryan reported that step counts of adults decreased by 3.6% for every 10 cm of snow on the ground [6]. Another previous study also demonstrated that physical activity decreases with aging and during winter in elderly Japanese people [7]. In the old age group, a decline in physical activity is associated with poor physical performance [8]. After controlling for age and/or gender, lower extremity function (walking speed and knee extension torque) showed significant improvement with an increase in the daily step count, particularly in individuals >75 years [8]. Few studies have examined whether change in physical activity in older persons during winter affected the level of their future physical performance.

The purpose of the present prospective study was to examine whether a change in physical activity in old-old women (age ≥ 75 years) living in northern Japan during winter affected the level of their physical performance. We hypothesized that those who maintained their daily step counts during the winter season would demonstrate better physical performance than those who had fewer step counts. We expect that the results of this study would promote the strategy to maintain the physical activity among old-old persons during winter.

## 2. Materials and Methods

### 2.1. Participants

As part of the “Population-Based and Inspiring Potential Activity for Old-Old Inhabitants (PIPAOI)” project, we conducted the present study in three phases (baseline: November 2012; 3-month follow-up: February 2013; 1-year follow-up: November 2013). The study was performed in Bibai, Hokkaido, located at the northern area of Japan. In 2012, Bibai's population was approximately 25,000 including 8,600 persons aged ≥ 65 years. The details of the PIPAOI project have been previously described in detail [9, 10]. The Ethics Committee of Sapporo Medical University Hospital approved the present study's protocol, and we conducted our study in accordance with the Declaration of Helsinki. Informed written consent was obtained from each participant prior to enrollment.

**Figure 1:**
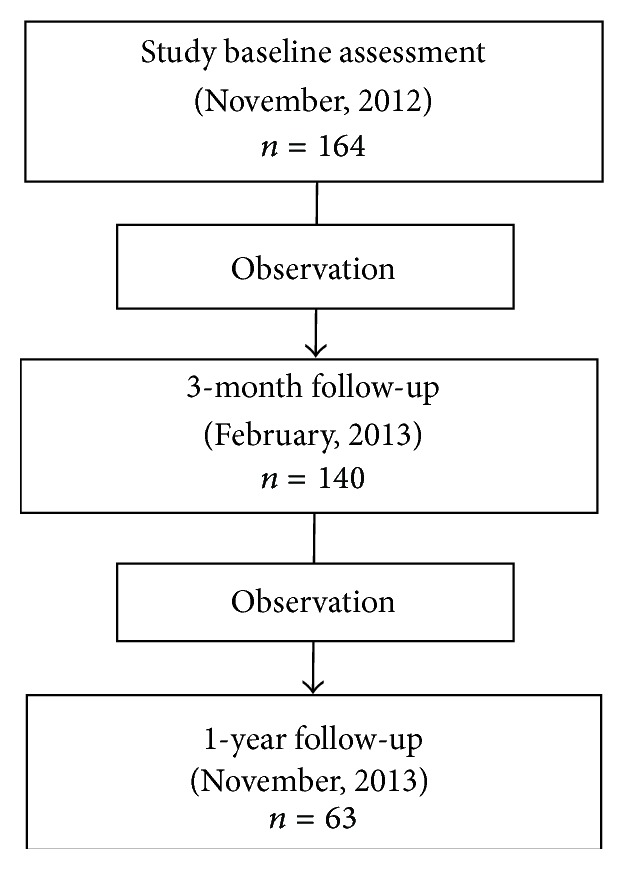
Participant flow.

An introductory letter was sent to approximately 800 community-dwelling elderly women. Eligible participants included women aged 75 years and older who were able to walk in their homes. One hundred and sixty-four eligible women participated in the baseline phase. Of these, 140 (85%) participated in the 3-month follow-up phase and 63 (45%) participated in the 1-year follow-up (Figure 1). Participants were excluded from the analysis if they were hospitalized for >1 week 3 months prior to the study because of high blood pressure, stroke, cardiovascular disease, respiratory disease, diabetes, joint pain, or osteoporosis.

### 2.2. Physical Activity Measurement

Each participant's physical activity was monitored using a Kenz Lifecorder GS device (Suzuken, Nagoya, Japan). The device was attached to the participant's buttock for 1 week. It tabulated the number of steps taken from the time the participant arose in the morning to the time she fell asleep at night. The data were uploaded and stored on a computer, and mean daily step counts were calculated. When body movement data were not recorded for >2 consecutive hours, the data for that day were excluded [11]. When the device was worn for less than three days in one week, the data for that week were excluded [12]. Step count data were obtained in November 2012 (baseline) and in February 2013 (3-month follow-up). Data from participants who decreased step counts between the baseline and 3-month follow-up rounds (declining group) were compared to the data of participants who maintained and improved step counts (maintenance group).

### 2.3. Physical Performance Measurement

The indicators of physical performance obtained included handgrip strength, knee extensor strength, static standing balance, hip walking distance, and maximum walking speed. These were assessed in November 2012 (baseline) and November 2013 (1-year follow-up). The best result obtained from each task was used for analysis of the physical performance tests.

#### 2.3.1. Handgrip Strength

Handgrip strength is regarded as a valid indicator of general health status [13]. One measurement of handgrip strength of the participant's dominant hand was obtained using a Smedley-type handheld dynamometer (Matsumiya Ika Seiki Mfg. Ltd., Tokyo, Japan).

#### 2.3.2. Knee Extensor Strength

Knee extensor strength was measured once by having the participant flex her knee 90° while seated on a chair. During the test, a testing pad was attached to the front lower leg of the participant and strapped to the leg of the chair [14]. The participant was instructed to use her leg to push the pad with maximum strength (N). In the data analysis, isometric knee extensor torque (Nm/kg) was normalized against arm moment (m) and body mass (kg).

#### 2.3.3. Total Length of the Center of Gravity

To assess balance (postural stability), postural sway during quiet standing was measured once using a force plate (ECG-1500A, KYOWA, Japan). Signals were sampled at 50 Hz and registered for a period of 20 s. The participant was instructed to keep her eyes open and to stand as symmetrically as possible. The center of pressure (COP) oscillation was determined using the total length of the participant's center of gravity, which indicated the amount of COP [15, 16].

#### 2.3.4. Hip Walking Distance

Hip walking distance was determined based on the results of an exercise used to train the muscles of the trunk [17]. For the hip walking distance test, the participant was sat on the floor and instructed to sit with outstretched legs and arms crossed over the chest; she was then instructed to move the buttocks forward as fast as possible. Trunk rotation by lifting the ischium was permitted. The distance that the participant moved forward within 10 s was recorded. The position of the participant's right lateral malleoli at the start of the exercise and after 10 s of hip walking was used as the starting and ending point for the distance measurement [18]. Each participant completed this test twice.

#### 2.3.5. Maximum Walking Speed

To measure maximum walking speed, an 11 m walkway was constructed, and the time required to walk 5 m was measured [19]. The participant was instructed to walk as fast as possible without running; she performed this task twice.

### 2.4. Other Covariates

For anthropometric measurements, participants were asked to wear light clothing and instructed to remove their shoes. Height (to the nearest 0.1 cm) and body mass (to the nearest 0.1 kg) were recorded. The subject's body mass index (BMI) was calculated using the standard formula: [weight (kg)]/[height (m)]^2^.

### 2.5. Statistical Analysis

Data were analyzed using SPSS version 20.0 (IBM Corp, Marmonk, NY, USA), with the significance level set at 5%. Differences between the declining group and the maintenance group were assessed using Student's *t*-test in baseline data. A general linear model for repeated-measures analysis of variance (ANOVA) was used to determine the group difference for the physical performance values. Two time points (baseline and 1-year follow-up) were treated as the within-subjects factor; the differences between the declining and maintenance groups were treated as the between-subjects factor.* Post hoc* comparisons were performed to assess the differences in physical performance variables between baseline and 1-year follow-up in each group.

## 3. Results

Thirteen women at baseline and 12 women during the 3-month follow-up period were unable to complete pedometer wearing as per a protocol. We obtained performance measures of 53 women whose step count data were obtained in the baseline and 3-month follow-up phases and who completed the physical performance tests in the baseline and 1-year follow-up phases. In this cohort, the mean age was 78.4 ± 3.2 years (Table 1). Thirty-one old-old women (58.5%) maintained the step counts during winter, while 22 women (41.5%) decreased the step counts. There were no significant differences in the baseline characteristics between the declining and maintenance groups (Table 2).

Table 2 depicts all physical performance variables for the declining and maintenance groups between baseline and 1-year follow-up. No interaction effects between group and time were detected for BMI and hip walking distance (*F*(1,51) = 0.65, *p* = 0.42 and *F*(1,49) = 0.36, *p* = 0.55, resp.). No interaction effects between group and time were detected for grip strength, knee extensor strength, and total center of gravity length (*F*(1,51) = 1.24, *p* = 0.27; *F*(1,50) = 1.45, *p* = 0.23; and *F*(1,49) = 0.44, *p* = 0.51, resp.).* Post hoc* comparisons revealed that the declining group had a significantly decreased grip strength after 1 year compared with the baseline (*p* = 0.02); however, no significant change was found for the maintenance group (*p* = 0.53). In addition,* post hoc* comparisons revealed that the maintenance group had a significantly increased knee extensor strength compared with the baseline (*p* = 0.0001); however, no significant increase was found for the declining group (*p* = 0.11). Both the declining and maintenance groups showed reduced total center of gravity length after 1 year (*p* = 0.02, *p* = 0.03, resp.). A statistically significant interaction effect between group and time was found for the maximum walking speed (*F*(1,50) = 5.23, *p* = 0.03).* Post hoc* comparisons revealed that the maintenance group significantly increased walking speed compared with the baseline (*p* = 0.01); however, no significant change was found for the declining group (*p* = 0.44).

## 4. Discussion

The goal of the present study was to measure any change in the physical activity of old-old women living in northern Japan during winter and to determine a difference in future physical performance by comparing groups of old-old women whose physical activity either declined or had no decrease.

We found that, among old-old women, the maintenance of physical activity in winter resulted in positive changes of knee extensor strength, total center of gravity length, and maximum walking speed after 1 year. We also found that a declining amount of physical activity in winter resulted in positive change in total center of gravity length in this age group as well as a negative change in grip strength after 1 year. Finally, we found a significant interaction effect between group and time in maximum walking speed.

These results showed that change in the level of physical activity by old-old women during winter has an influence on their physical performance after 1 year.

Numerous studies have shown that physical activity has a cross-sectional association with physical performance among older adults. After controlling data for age and/or gender, lower extremity function (walking speed and knee extension torque) showed a significant positive relationship with daily step counts, particularly in individuals >75 years [8]. In another study, inactive individuals had poorer scores on lower extremity performance than individuals with an active lifestyle [20]. In multiple regression analyses, moderately vigorous physical activity was found to be associated with the Short Physical Performance Battery (SPPB) summary score [21]. An earlier study of a randomized controlled trial in sedentary old people reported that a structured physical activity intervention improved the SPPB score and 400 m walking speed [22].

Although an association between physical activity and physical performance has been shown, the effect of a change in the physical activity level during winter on physical performance had not been found. The results of our study showed that even a short-term change in the level of physical activity had an influence on later physical performance. Therefore, although the health status of individuals who maintained their physical activity level in winter may remain stable and relate to better future physical performance, it is possible that predicting a performance decline over time can be made for those individuals whose winter activity level decreased.

Maximum walking speed has been shown as one of the most important indexes for predicting movement performance ability in community-dwelling old individuals [23]. It is also associated with the future decline of instrumental activities of daily living (IADL) [24] and is also linked to a risk of all-cause mortality [25]. Because the present study demonstrated that a change in the physical activity level during winter influences maximum walking speed after 1 year, future study would be required to determine what kind of intervention is most effective to maintain physical activity in winter.

In both groups, we found that after one year there were positive changes in knee extensor strength. Because knee extensor strength is a measurement that is not to be used in elderly subjects, the learning and measuring effects may have influenced the repeated measurements.

There are certain limitations of the present study. First, the 1-year follow-up sample was much smaller than the baseline sample, and the effect of the changes in physical activity may not be sufficiently clear. In studies involving pedometer wearing, many subjects are excluded from the protocol. The elderly women who completed this study had a high functional capacity and were able to complete examination three times and the pedometer wearing two times. The elderly women who dropped out were more likely to be frail compared with the subjects who completed this study. Therefore, the effect of maintenance of physical activity during winter season may be clearer. Second, the participants in our study were all women; therefore, we cannot extrapolate the results to elderly men. Third, the participants' activity and performance were only followed for 1 year. Further long-term follow-up studies should be conducted. Fourth, colds and physical illness may influence changes in physical activity during winter season as well as hospitalization. These need to be taken into consideration in future studies related to physical activity during winter.

## 5. Conclusions

The present study showed that a change of physical activity during winter has an influence on the physical performance after 1 year among community-dwelling old-old women. In particular, the change of physical activity during winter was shown to affect the maximum walking speed even a year later. In the future, it is necessary that elderly individuals maintain an adequate level of physical activity during winter to optimize their physical performance.

## Figures and Tables

**Table 1:** Subject characteristics.

	Total (*n* = 53)
Age (years)	78.4 ± 3.2
Height (cm)	148.5 ± 6.0
Weight (kg)	50.8 ± 7.7
BMI (kg/m^2^)	23.0 ± 3.1
Step count (steps/day)	
Baseline (November, 2012)	5466.9 ± 3044.2
3-month follow-up (February, 2013)	5735.4 ± 3091.1
Decline of step counts (3-month follow-up, baseline)	22 (41.5)

Mean ± SD, numbers (%).

**Table 2:** Change of the physical performance between baseline and 1-year follow-up.

Measure	Group	Baseline	1-year follow-up	*Post hoc* test	Time effect		Time × group *interaction *	
				*p *	*F* ^a^	*p *	*F* ^a^	*p *
Age (years)	Maintenance (*n* = 31)	78.4 ± 3.2						
	Declining (*n* = 22)	78.3 ± 2.6						

BMI (kg/m^2^)	Maintenance (*n* = 31)	23.0 ± 3.1	22.8 ± 3.1	0.18	6.57	0.01	0.65	0.42
	Declining (*n* = 22)	23.2 ± 3.3	22.7 ± 3.8	0.04				

Grip strength (kg)	Maintenance (*n* = 31)	22.8 ± 4.4	22.1 ± 4.3	0.53	4.01	0.05	1.24	0.27
	Declining (*n* = 21)	22.7 ± 3.8	21.5 ± 4.6	0.02				

Knee extensor strength (Nm/kg)	Maintenance (*n* = 30)	1.02 ± 0.28	1.17 ± 0.29	0.0001	18.27	0.0001	1.45	0.24
	Declining (*n* = 22)	0.98 ± 0.28	1.09 ± 0.31	0.11				

Total length of center of gravity with eyes open (cm)	Maintenance (*n* = 30)	66.7 ± 11.3	63.8 ± 9.2	0.02	13.81	0.0005	0.44	0.51
	Declining (*n* = 21)	64.3 ± 10.7	60.3 ± 12.2	0.01				

Hip walking distance (cm)	Maintenance (*n* = 30)	78.7 ± 26.0	78.3 ± 28.8	0.59	0.00	0.97	0.36	0.55
	Declining (*n* = 21)	70.7 ± 21.8	72.3 ± 23.7	0.75				

Maximum walking speed (m/s)	Maintenance (*n* = 30)	1.71 ± 0.28	1.75 ± 0.26	0.01	0.82	0.37	5.23	0.03
	Declining (*n* = 22)	1.74 ± 0.33	1.71 ± 0.32	0.44				

Mean ± SD.

^a^Repeated measures ANOVA.
